# Extensively drug-resistant *Alcaligenes faecalis* infection

**DOI:** 10.1186/s12879-020-05557-8

**Published:** 2020-11-11

**Authors:** Chienhsiu Huang

**Affiliations:** Department of Internal Medicine, Division of Chest Medicine, Dalin Tzu Chi Hospital, Buddhist Tzu Chi Medical Foundation, NO. 2, Min-Sheng Road. Dalin Town, Chiayi County, Taiwan

**Keywords:** *Alcaligenes faecalis* infection, Extensively drug-resistant

## Abstract

**Background:**

*Alcaligenes faecalis* is usually causes opportunistic infections in humans. *Alcaligenes faecalis* infection is often difficult to treat due to its increased resistance to several antibiotics. The results from a clinical study of patients with *Alcaligenes faecalis* infection may help improve patients’ clinical care.

**Methods:**

We conducted a retrospective analysis of all patients presenting with *Alcaligenes faecalis* infection from January 2014 to December 2019. The medical records of all patients were reviewed for demographic information, clinical symptoms and signs, comorbidities, use of intravenous antibiotics within the past three months, bacterial culture, antibiotics sensitivity test, and clinical outcomes.

**Results:**

Sixty-one cases of *Alcaligenes faecalis* infection were seen during the study period, including 25 cases of cystitis, nine cases of diabetic foot infection, eight cases of pneumonia, seven cases of acute pyelonephritis, three cases of bacteremia, and nine cases of infection at specific sites. Thirty-seven patients (60.7%) had a history of receiving intravenous antibiotics within three months of the diagnosis. Fifty-one (83.6%) cases were mixed with other bacterial infections. Extensively drug-resistant infections have been reported since 2018. The best sensitivity rate to *Alcaligenes faecalis* was 66.7% for three antibiotics (imipenem, meropenem, and ceftazidime) in 2019. Two antibiotics (ciprofloxacin and piperacillin/tazobactam) sensitivity rates to *A. faecalis* were less than 50%.

**Conclusions:**

The most frequent *Alcaligenes faecalis* infection sites, in order, are the bloodstream, urinary tract, skin and soft tissue, and middle ear. The susceptibility rate of *Alcaligenes faecalis* to commonly used antibiotics is decreasing. Extensively drug-resistant *Alcaligenes faecalis* infections have emerged.

**Supplementary Information:**

The online version contains supplementary material available at 10.1186/s12879-020-05557-8.

## Introduction

*Alcaligenes faecalis* (*A. faecalis*) is a Gram-negative, obligate aerobic, oxidase-positive, catalase-positive, and nonfermenting bacterium. It is commonly found in soil, water, and in hospital settings, such as in respirators, hemodialysis systems, and intravenous solutions [1, 2]. It is a potentially emerging pathogen and usually causes opportunistic infections in humans. The organism has been isolated from a range of clinical materials, such as urine, blood, wound discharge, stool, cerebrospinal fluid, and respiratory secretions [3–6]. *A. faecalis* has been associated with endocarditis, bacteremia, meningitis, endophthalmitis, skin and soft tissue infections, urinary tract infections, otitis media, peritonitis, and pneumonia [1, 2, 7–28]. *A. faecalis* infection is often difficult to treat due to its increased resistance to several antibiotics, such as anti-pseudomonas penicillin, cephalosporins, carbapenems, aminoglycosides, and quinolones [17, 23, 24, 26]. Optimal antibiotic therapy for *A. faecalis* has not been well established in the literature. We report the results of a clinical study of patients with *A. faecalis* infection in this article. This study aims to emphasize the emergence of extensively drug-resistant *A. faecalis* and to provide local susceptibility data for the same.

## Methods

### Study design

We conducted a retrospective analysis of all patients presenting with *A. faecalis* infection who were admitted to Dalin Tzu Chi Hospital from January 2014 to December 2019. Patients were diagnosed with *A. faecalis* infection when their clinical symptoms and signs indicated infection.

### Data collection

The data were obtained from the hospital’s clinical information system, microbiology laboratory report system, and medical chart review. We obtained details on all *A. faecalis* infections (including community-onset infection and hospital-acquired infection), patient demographics, clinical symptoms and signs, details of hospital course, comorbidities, prior intravenous antibiotic use within 90 days, bacterial cultures, antibiotics sensitivity tests, and clinical outcomes.

### Antibiotic susceptibility

Antibiotic susceptibility was tested using the VITEK® II system with VITEK® II Gram Negative Susceptibility cards (bioMérieux, Marcy-l’Étoile, France) with Clinical & Laboratory Standards Institute interpretive criteria M100–25. One Gram-negative (GN) identification card and another VITEK II AST-N322 card (for susceptibility testing of aerobic GN bacilli against specified antimicrobials) were placed in the neighboring slots, along with the transfer tube and the corresponding suspension tube [29]. The complete list of antibiotics used in susceptibility testing for *A. faecalis*, including piperacillin, piperacillin-tazobactam, ceftazidime, cefepime, cefotaxime, ceftriaxone, ampicillin-sulbactam, imipenem, meropenem, gentamicin, amikacin, ciprofloxacin, levofloxacin in our microbiology laboratory which was reference from clinical and laboratory standards institute (30th edition). Our microbiology laboratory susceptibility test report for *A. faecalis* included piperacillin-tazobactam, ceftazidime, cefepime, imipenem, meropenem, gentamicin, amikacin, ciprofloxacin, and ampicillin-sulbactam. If the susceptibility test showed resistant to all antibiotics, tigecycline susceptibility in *A. faecalis* would be done by the disc diffusion method.

We also searched PubMed and google scholar for topics related to *Alcaligenes faecalis* with no language restrictions.

The definition of MDR/XDR cites the literature of Maggiorakos et al. [30]

### Study ethics and consent procedure

Our study was a retrospective analysis of medical records. The six-year study represented the lowest risk to the research subjects, and all information was made anonymous before being made available for research. The project was approved by the Buddhist Dalin Tzu Chi General Hospital Research Ethics Committee (Approved IRB No. B10802024), which exempted the study from the requirement for informed consent.

### Statistical analysis

Continuous variables were expressed as mean ± standard deviation or median (range), whereas categorical variables were expressed as frequencies and percentages. The trend in change of antimicrobial susceptibility analyzed by linear-by-linear association in chi-square test. All statistical analyses were conducted using the statistical package SPSS for Windows (Version 17.0, SPSS, Inc., Chicago, IL, USA). A *p*-value < 0.05 was considered statistically significant.

## Results

Sixty-one cases of *A. faecalis* infection were identified during the study, including 25 cases of cystitis, nine cases of diabetic foot ulcer with infection, eight cases of pneumonia, seven cases of acute pyelonephritis (APN), three cases of bacteremia, and nine cases of infection at specific sites. There were nine hospital-acquired infections cases, including three cases of diabetic foot ulcer with infection, two cases of pneumonia, one case of pleural empyema, one case of peritonitis, one case of surgical wound infection, and one case of leg burn wound infection. Thirty-one community-onset infection cases (31/52 = 59.6%) had a history of previous hospitalisation within 90 days. Thirty-seven patients (60.7%) had a history of intravenous antibiotic use within the past 90 days. The most commonly used antibiotics were ceftazidime (11 cases) and levofloxacin (ten cases). There were only two cases of receipt of antibiotics during the same hospitalisation but before the onset of *A. faecalis* infection, including one case of prolonged mechanical ventilation with pneumonia and one case of peritonitis. The length of stay before *A. faecalis* isolation of the two cases were 190 days and 22 days, respectively. Fifty-one (83.6%) cases were mixed with other bacterial infections. The most common mixed infection pathogens were Enterococcus species (nine cases), Proteus vulgaris (nine cases), and *Pseudomonas aeruginosa* (eight cases).

### Cystitis with *A. faecalis* (Table 1)

Twenty-five cases of *A. faecalis* cystitis were seen during the study period, which accounts for 41% of *A. faecalis* infections in this report. There were ten females and 15 males in our study. The mean age was 76.9 years (range: 25 to 98 years). Sixteen cases (64%) were catheter related cystitis. One patient had no comorbidity, while 24 patients had comorbidities such as diabetes mellitus, hypertension, dementia, cerebrovascular accident and chronic kidney disease (supplement). The most common was neurologic comorbidity (18 cases). Fourteen patients had a history of intravenous antibiotic exposure within the past 90 days. Nineteen patient urine cultures displayed polymicrobial infection. The most common mixed infectious pathogen was Providencia species (five cases). The antibiotics sensitivity test showed no presence of extensively drug-resistant (XDR) *A. faecalis*. Two patients died from urosepsis. Twenty-three patients were treated with adequate antibiotics therapy and were discharged in excellent condition.

**Table 1:** Bacteriology and clinical outcome in twenty-five cases of *Alcaligenes faecalis* infection cystitis

	Case 1	Case2	Case 3	Case 4	Case 5	Case 6	Case7	Case8	Case9
year	2014	2014	2014	2014	2014	2014	2015	2015	2015
catheter related cystitis	No	No	Yes	Yes	Yes	Yes	Yes	Yes	Yes
prior intravenous antibiotic use within 90 days	No	No	No	No	FEP	unknown (other hospital)	No	No	No
Antibiotic therapy	MXF	LVX	LVX SAM	FEP LVX	ETP	CTX	FEP	MXF	CAZ
outcome	cure	cure	cure	cure	cure	dead	dead	cure	cure
Results of antibiotics sensitivity test of *Alcaligenes faecalis*									
GEN	S	S	S	S	S	S	S	S	R
AMK	S	S	S	S	S	S	S	S	R
CAZ	S	S	S	S	S	S	S	S	S
FEP	S	S	S	S	R	R	S	S	R
SAM	S	S	S	S	S	S	S	S	R
TZP	S	S	S	S	S	S	S	S	S
CIP	S	S	R	S	R	R	S	S	R
IPM	S	S	S	S	S	S	S	S	S
MEM	S	S	S	S	S	S	S	S	S
TGC	NA	NA	NA	NA	NA	NA	NA	NA	NA
Mixed infection pathogens									
Enterococcus spp			V						
Proteus vulgaris							V		
Citrobacter koseri								V	
Klebsiella pneumoniae						V			
Providencia rettgeri					V	V			
Non-fermentative GNB	V								
	Case 10	Case 11	Case 12	Case 13	Case 14	Case 15	Case 16	Case 17	
year	2015	2015	2015	2016	2016	2016	2016	2016	
catheter related cystitis	Yes	No	No	No	Yes	No	Yes	No	
prior intravenous antibiotic use within 90 days	No	LVX	CAZ CRO	LVX	LVX	NO	No	No	
Antibiotics therapy	CMZ	SAM	LVX	ETP	CAZ	LVX	LVX	ETP	
outcome	cure	cure	cure	cure	cure	cure	cure	cure	
Results of antibiotics sensitivity test of Alcaligenes faecalis									
GEN	S	S	S	R	S	NIL	NIL	S	
AMK	S	S	S	S	S	NIL	NIL	S	
CAZ	S	S	S	S	S	NIL	NIL	S	
FEP	S	S	S	S	S	NIL	NIL	S	
SAM	S	S	S	S	S	NIL	NIL	S	
TZP	S	R	S	R	S	NIL	NIL	R	
CIP	S	R	S	R	R	NIL	NIL	R	
IPM	S	S	S	S	S	NIL	NIL	S	
MEM	S	S	S	S	S	NIL	NIL	S	
TGC	NA	NA	NA	NA	NA	NA	NA	NA	
Mixed infection pathogens									
Chryseobacterium meningosepticum		V							
Serratia marcescens						V			
Providencia stuartii					V			V	
Escherichia coli	V			V					
	Case18	Case 19	Case20	Case21	Case 22	Case 23	Case 24	Case 25	
year	2016	2017	2017	2017	2018	2018	2018	2019	
catheter related cystitis	Yes	No	Yes	No	Yes	Yes	Yes	Yes	
prior intravenous antibiotic use within 90 days	CRO CFZ	Unknow (other Hospital)	LVX	CFZ CMZ	LVX FEP	Unknow (other Hospital)	FEP TEC	Unknow (other Hospital)	
Antibiotics therapy	SAM	LVX	CAZ	CAZ	LVX	MEM	FEP	FEP	
outcome	cure	cure	cure	cure	cure	cure	cure	cure	
Results of antibiotics sensitivity test of Alcaligenes faecalis									
GEN	S	S	S	S	S	S	S	S	
AMK	S	S	S	S	S	S	S	S	
CAZ	S	S	S	S	S	S	S	S	
FEP	S	S	S	S	S	S	S	S	
SAM	S	S	S	S	S	S	S	S	
TZP	R	S	R	R	S	R	R	R	
CIP	R	S	R	R	S	R	R	S	
IPM	S	S	S	S	S	S	S	S	
MEM	S	S	S	S	S	S	S	S	
TGC	NA	NA	NA	NA	NA	NA	NA	NA	
Mixed infection pathogens									
Proteus vulgaris								V	
Enterobacter cloacae	V								
*Pseudomonas aeruginosa*		V							
Providencia stuartii							V		
Morganella morganii			V						
Enterococcus					V				
Serratia marcescens				V					

### Acute pyelonephritis (Table 2)

Seven patients (six females and one male) with *A. faecalis* acute pyelonephritis were seen during the study. The mean age was 75.4 years (range: 63 to 85 years). All seven patients had a risk of obstructive uropathy (four cases of cervical carcinoma, two cases of renal stones, and one case of right ureteral cancer). Four patients had a history of intravenous antibiotic exposure within the past 90 days. Six patient urine cultures displayed polymicrobial infection. The most common mixed infectious pathogen was Providencia rettgeri, which affected three patients. The antibiotic sensitivity test showed no presence of XDR *A. faecalis*. All patients received adequate IV antibiotic therapy, and all were discharged from the hospital in excellent condition.

**Table 2:** Bacteriology and clinical outcome in seven cases of Alcaligenes faecalis acute pyelonephritis

	Case 1	Case 2	Case 3	Case 4	Case 5	Case 6	Case7
year	2014	2014	2015	2015	2016	2017	2017
prior intravenous antibiotic use within 90 days	No	Unknown (other hospital)	no	ETP LVX CMZ	No	ETP TEC CMZ	AMC ETP CFZ
Antibiotics therapy	SAM	CIP	CIP	LVX	ETP	ETP	TEC ERT
outcome	cure	cure	cure	cure	cure	cure	cure
Results of antibiotics sensitivity test of Alcaligenes faecalis							
GEN	S	S	S	S	S	S	S
AMK	S	S	S	S	S	S	S
CAZ	S	S	S	S	S	S	S
FEP	S	S	S	S	S	S	S
SAM	S	S	S	S	S	S	S
TZP	S	S	S	S	S	R	S
CIP	R	S	S	S	R	R	R
IPM	S	S	S	S	S	S	S
MEM	S	S	S	S	S	R	S
TGC	NA	NA	NA	NA	NA	NA	NA
Mixed infection pathogens							
Proteus vulgaris					V		V
Escherichia coli					V		
Klebsiella pneumoniae						V	
Providencia rettgeri		V	V			V	
enterococcus				V			

### Diabetic foot ulcer with *A. faecalis* infection (Table 3)

Six male patients and three female patients had diabetic foot ulcers with *A. faecalis* infection. The mean age was 57.2 years (range: 41 to 85 years). All patients had other comorbidities. All patients’ foot ulcer lesions were chronic (range: 14 days to 18 months). Two patients had no history of prior intravenous antibiotic use within 90 days. All patients’ wound cultures displayed polymicrobial infection. The antibiotics sensitivity test showed the presence of XDR *A. faecalis* infection beginning in 2019. All patients required surgical intervention. The wounds did not heal in three patients.

**Table 3:** Bacteriology and clinical outcome in nine cases of diabetic foot ulcer with Alcaligenes faecalis infection

	Case 1	Case2	Case 3	Case 4	Case 5	Case 6	Case7	Case8	Case9
year	2014	2015	2015	2017	2018	2018	2019	2019	2019
prior intravenous antibiotic use within 90 days	CIP SAM	PIP CRO VAN	No	CFZ TEC	No	CAZ CIP PIP TEC	CAZ VAN	LVX MEM TEC	CRO CAZ OXA
Antibiotic therapy	SAM	MEM VAN	VAN CAZ	OXA CMZ	MEM VAN	CAZ TEC	CAZ VAN	CST	CAZ MEM
outcome	heal	heal	heal	heal	no heal	no heal	heal	no heal	heal
Results of antibiotics sensitivity test of Alcaligenes faecalis									
GEN	S	S	S	R	S	I	R	R	S
AMK	S	S	S	S	S	S	R	R	S
CAZ	S	S	S	S	S	S	R	R	S
FEP	S	S	S	I	S	I	R	R	S
SAM	S	S	S	I	S	S	R	R	S
TZP	S	S	S	I	S	S	R	R	R
CIP	S	S	S	R	S	R	R	R	S
IPM	S	S	S	S	S	S	R	R	S
MEM	S	S	S	S	S	S	R	R	S
TGC	NA	NA	NA	NA	NA	NA	S	S	NA
Mixed infection pathogens									
Enterococcus spp	V				V			V	V
Proteus vulgaris		V			V				
Citrobacter koseri			V						
MRSA			V						
Klebsiella pneumoniae						V	V	V	
Pseudomonas aeruginosa									V
Providencia rettgeri						V			
Acinetobacter baumannii							V	V	
Morganella morganii				V					

### Pneumonia (Table 4)

Eight cases (six males, two females) of *A. faecalis* pneumonia were seen during the study period. The mean age was 70.0 years (range: 51–83 years). All eight patients were at risk of pneumonia (three patients had malignancies, one had end-stage renal disease, and four patients were bed-ridden with neurologic deficits). One patient had no history of prior intravenous antibiotic use within 90 days. Six patient sputum cultures displayed polymicrobial infection. The antibiotics sensitivity test showed the presence of XDR *A. faecalis* beginning in 2018. Two patients died from *A. faecalis* pneumonia. Six patients received adequate intravenous antibiotics therapy and were discharged in excellent condition.

**Table 4:** Bacteriology and clinical outcome in eight cases of Alcaligenes faecalis pneumonia

	Case 1	Case 2	Case 3	Case 4	Case 5	Case 6	Case7	Case8
year	2014	2014	2015	2018	2019	2019	2019	2019
prior intravenous antibiotic use within 90 days	No	ETP FEP VAN	LVX	CAZ LVX DOR VAN	CAZ AMK IPM TEC CIP	TZP MEM CAZ TEC AMK	CRO SAM	TZP MEM CAZ VAN LVX AMK
Antibiotics therapy	CXM	CRO	FEP	TGC	TGC	MEM	CAZ	CAZ
outcome	cure	cure	dead	cure	cure	cure	dead	cure
Results of antibiotics sensitivity test of Alcaligenes faecalis								
GEN	S	S	R	R	R	R	S	NIL
AMK	S	S	R	R	R	R	S	NIL
CAZ	S	S	R	R	R	S	S	NIL
FEP	S	S	R	R	R	R	S	NIL
SAM	S	S	R	R	R	R	S	NIL
TZP	S	S	R	R	R	S	R	NIL
CIP	S	S	R	R	R	R	R	NIL
IPM	S	S	S	R	R	S	S	NIL
MEM	S	S	S	R	R	S	S	NIL
TGC	NA	NA	NA	S	S	NA	NA	NA
Mixed infection pathogens								
Proteus vulgaris	V							
MSSA							V	
Pseudomonas aeruginosa						V		V
Providencia stuartii		V						
Brukholderia cepacia			V					

### Bacteremia (no concurrent primary site of infection)

Three patients developed bacteremia during the study period. These three patients included a 78-year-old male, a bedridden stroke survivor; an 81-year-old male, a bedridden dementia; and an 81-year-old female with cholangiocarcinoma and dementia. The two male patients had a history of intravenous antibiotic exposure within the past 90 days. Two patients’ blood cultures displayed polymicrobial infection (one patient had a mixed infection with *Enterococcus faecalis*, and another patient had a mixed infection with Morganella morganii). Antibiotic sensitivity tests showed no presence of XDR *A. faecalis*. All patients received adequate antibiotics therapy and were discharged in excellent condition. Nine specific sites of *A. faecalis* infection cases are shown in Table 5. The trend in change of antimicrobial susceptibility analyzed by linear-by-linear association in chi-square test (Table 6). The susceptibility rate of *A. faecalis* to commonly used antibiotics (except ciprofloxacin and cefepime) is decreasing year by year.

**Table 5:** Bacteriology and clinical outcome in nine cases with Alcaligenes faecalis infection

	Case 1	Case2	Case 3	Case 4	Case 5	Case 6	Case7	Case8	Case9
year	2016	2018	2017	2019	2015	2016	2016	2016	2019
prior intravenous antibiotic use within 90 days	NIL	AMC	No	No	CAZ CIP MEM	No	No	CFZ	No
Antibiotics therapy	MXF AMC	CAZ	OFX	SXT	CAZ	CFZ	CTB	CFZ	TEC CRO
outcome	cure	cure	cure	cure	cure	cure	cure	cure	cure
Results of antibiotics sensitivity test of Alcaligenes faecalis									
GEN	R	S	S	S	S	R	NIL	NIL	S
AMK	S	S	S	S	S	S	NIL	NIL	S
CAZ	R	S	S	S	S	R	NIL	NIL	S
FEP	R	S	S	S	S	R	NIL	NIL	S
SAM	R	S	S	S	S	R	NIL	NIL	S
TZP	R	S	S	S	S	R	NIL	NIL	S
CIP	R	S	S	R	R	S	NIL	NIL	S
IPM	S	S	S	S	S	S	NIL	NIL	S
MEM	S	S	S	S	S	S	NIL	NIL	S
TGC	NA	NA	NA	NA	NA	NA	NA	NA	NA
Mixed infection pathogens									
Enterococcus									V
Proteus vulgaris								V	V
Enterobacter cloacae		V			V				
Pseudomonas aeruginosa	V		V					V	V
Acinetobacter baumannii		V							
Escherichia coli							V		
Serratia marcescens				V					
Providencia stuartii	V								
MSSA				V					
Streptococcus group D							V		
Aeromonas hydrophila						V			

Case 1 and case 2: Pleural empyema, Case 3 and case 4: otitis media, Case 5: peritonitis, Case 6: surgical wound infection,

Case 7: pelvis abscess, Case 8: scrotum abscess, Case 9: burn wound infection

**Table 6:** The trend in change of antimicrobial susceptibility to Alcaligenes faecalis analyzed by linear-by-linear association in chi-square test

Antibiotics/year (number of patients)	2014 (12)	2015 (13)	2016 (9)	2017 (9)	2018 (7)	2019 (9)	M^2^	*P* value
CAZ	100%	92.3%	77.8%	88.7%	85.7%	66.7%	4.063	0.044
GEN	100%	84.6%	55.6%	77.8%	71.4%	55.6%	5.144	0.023
AMK	100%	84.6%	100%	100%	85.7%	55.6%	5.837	0.016
CIP	50.0%	61.5%	22.2%	22.2%	42.9%	33.3%	1.589	0.207
IMP	100%	100%	100%	100%	85.7%	66.7%	9.042	0.003
SAM	100%	84.6%	77.8%	88.7%	85.7%	55.6%	4.622	0.032
FEP	83.3%	76.9%	77.8%	66.7%	71.4%	55.6%	2.028	0.154
MEM	100%	100%	100%	100%	85.7%	66.7%	9.042	0.003
TZP	100%	84.6%	33.3%	42.4%	57.1%	33.3%	11.713	0.001

## Discussion and literature review

According to the literature, there were 130 sporadically reported cases of *A. faecalis* infection (Table 7) [1, 2, 7–28, 31–46]. The most commonly reported cases involved bacteremia, and most cases occurred in newborns and infants. In 1960, Doxiadis reported 33 cases of bacteremia in newborns, which was the largest case series of *A. faecalis* bacteremia [40]. *A. faecalis* was resistant to sulfonamides, and there were 20 deaths due to *A. faecalis* bacteremia. Fillipe reported 20 cases of chronic otitis media in Angola [19]. The use of bird feces by residents as a traditional remedy to prevent ear discharge was related to these *A. faecalis* chronic otitis media cases. The other infections from *A. faecalis* that have been reported in prior studies, in order of occurrence, were meningitis, skin and soft tissue infection (SSTI), and UTI. In our series, the most frequent cases were, in order of occurrence, UTI, SSTI, and pneumonia. The cases reported in the literature and our cases indicate that the most frequent *A. faecalis* infection sites, in order, are the bloodstream, urinary tract, skin and soft tissue (diabetic foot ulcer accounts for 56.5% of skin and soft tissue infections), and middle ear.

**Table 7:** Alcaligenes faecalis infection cases

Year/diagnosis	Cases of literature	Our cases	total
Cystitis	11	25	36
Skin and soft tissue infection	12	11*	23
Pneumonia	7	8	15
Acute pyelonephritis	0	7	7
Bacteremia	42	3	45
Pleural empyema	0	2	2
Otitis media	21	2	23
Meningitis	16	0	16
Endocarditis	3	0	3
Ocular infection	10	0	10
Peritonitis	2	1	3
Infectious diarrhea	3	0	3
specific sites infection	3	2	5
Total	130	61	191

*: including 9 cases of diabetic foot infection, 1 case of surgical wound infection and 1 case of burn wound infection

### *A. faecalis* isolation in mixed culture as a pathogen or contaminant

In Tena’s report, two out of five skin and soft tissue *A. faecalis* cases were mixed with other bacterial infections [17]. In Filipe’s series, all 20 *A. faecalis* otitis media cases were mixed with other bacterial infections [19]. Kahveci reported a case of *A. faecalis* peritonitis and concluded that it was important to view *A. faecalis* as a pathogen rather than a contaminant [15]. Junejo notes that it is evident that any organism found in the culture should not be completely disregarded and marked as a contaminant [23].. Al-Zakhari explains that serious illnesses and even death can be caused by *A. faecalis*. Therefore, rather than a contaminant, *A. faecalis* should be regarded as a pathogen, because global cases of life-threatening infections caused by *A. faecalis* are emerging [27]. In 2017, Laham reported a clinical sample study of an *A. faecalis* strain isolated from two outpatients and three inpatients, including four wounds cultures and one urine culture [47]. However, this study was only conducted for a three-month period. In 2013, Khajuria reported a total of 15 clinical isolates of *A. faecalis* specimens such as urine, pus, blood, and body fluids [48]. We believe that many cases of *A. faecalis* infections cases exist but have not been reported in the literature. Our series of *A. faecalis* infection cases were about 10 cases every year, which was only a small fraction of the infectious diseases in our hospital. We concur with Junejo and believe *A. faecalis* to be an infectious pathogen rather than contaminant. In our series, the majority of cultures were mixed with other well-established pathogens. However, one should consider that *A. faecalis* may be a contaminant in some cases, particularly for those who were cured despite lack of active treatment.

### The trend of antibiotic sensitive rate of *A. faecalis*

In 1997, Bizet first reported that *A. faecalis* strains were resistant to amoxicillin, ticarcillin, and gentamicin [1]. Amoxicillin-clavulanic acid and cefotaxime provided a successful treatment outcome for patients with *A. faecalis* infection. In 2000, Pereira reported that a strain of *A. faecalis* resistant to expanded-spectrum beta-lactamase cephalosporins was isolated from the urine of an inpatient [2]. In 2005, Dubois described the isolation of *A. faecalis* with ESBL in a patient with a concurrent urinary tract infection [11]. In 2017 and 2018, two cases of XDR *A. faecalis* pneumonia were reported by Agarwal and Junejo [22, 23]. In 2019, Hasan reported that a 60-year-old female with pandrug-resistant *A. faecalis* bacteremia who was treated with double-dose tigecycline had a successful treatment outcome [26]. Three articles on pandrug-resistant *A. faecalis* were published in 2020. Al-Zakhari reported a fatal case of cavitary pneumonia caused by pandrug-resistant *A. faecalis*. The patient died despite the aggressive antibiotic treatment (linezolid and polymyxin B) [27]. Majewski reported a pandrug-resistant *A. faecalis* hospital acquired urinary tract infection patient; the patient died in hospital [28]. Ngbede identified mobile colistin resistance genes in *Alcaligenes faecalis* from human clinical samples [49].

In March 2015, the strain *A. faecalis* exhibited sensitivity only to imipenem and meropenem in a pneumonia patient in our hospital. In May 2018, a strain of XDR *A. faecalis* susceptible only to tigecycline was isolated from a pneumonia patient. There were four cases with XDR *A. faecalis* infection in our series, including two cases of pneumonia and two cases of diabetic foot infection.

In view of individual antibiotics, ciprofloxacin revealed a very low susceptibility rate of *A. faecalis* from 2014 to 2019. Piperacillin/tazobactam was effective in significantly decreasing the susceptibility rate of *A. faecalis* since 2016. Emerging resistant strains of *A. faecalis* to imipenem and meropenem have been found since 2018. A high resistance rate of many antibiotics was also found in 2019. The best sensitivity rate to *A. faecalis* was 66.7% for three antibiotics (imipenem, meropenem, and ceftazidime). In two antibiotics (ciprofloxacin and piperacillin/tazobactam) sensitivity rates to *A. faecalis* were less than 50%.

Based on our prior experience, we selected an appropriate antibiotic for a susceptible *A. faecalis* infection patient according to the results of his or her antibiotics sensitivity test. If the *A. faecalis* organism is an ESBL strain, carbapenem is an appropriate antibiotic. Four articles mentioned that *A. faecalis* is susceptible to colistin [8, 19, 22, 23]. Data was not available regarding *A. faecalis* susceptibility to colistin in this study. If the *A. faecalis* is an XDR strain, we recommend that tigecycline is effective to XDR *A. faecalis*.

### Treatment failure of *A. faecalis* infection cases

Among our reported cases of *A. faecalis* infection, there were seven treatment failure cases, including two cases of pneumonia, two cases of cystitis complicated with sepsis, and three cases of diabetic foot infection. The overall treatment failure rate was 11.5%. *A. faecalis* is a low virulence bacterium. With adequate intravenous antibiotic therapy, patients with *A. faecalis* infection will typically experience a positive treatment outcome.

### Patients were cured with non-covering regimens

Four cases were cured with non-covering antibiotics, including two cases of diabetic foot infection, one case of pleural empyema, and one case of surgical wound infection. The four cases had received appropriate wound care, adequate abscess drainage, and surgical intervention which may be crucial for curing of infections.

### Limitations

Our clinical study of *A. faecalis* infection was a small case series and therefore can provide only minimal clinical experience. Additional case series reports of *A. faecalis* infection will add to the knowledge of how to treat *A. faecalis* infection. Misidentification of Acinetobacter baumannii as *Alcaligenes faecalis* by VITEK II system was reported in the literature [50]. Matrix-assisted laser desorption ionization time of flight mass spectrometry and 16S rRNA sequencing are helpful for the accurate identification between these two species [51]. However, we cannot perform the two methods to confirm the isolates in our hospital. If the culture is XDR *A. faecalis*, matrix-assisted laser desorption ionization time of flight mass spectrometry and 16S rRNA sequencing can be performed. An accurate distinction between Acinetobacter baumannii and *A. faecalis* has substantial clinical significance.

## Conclusions

*A. faecalis* exhibited decreasing sensitivity rate to commonly used antibiotics in 2019. Extensively drug-resistant *Alcaligenes faecalis* infections have emerged recently. We select an antibiotic for patients susceptible to *A. faecalis* infection based on the results of the antibiotics sensitivity test. With adequate intravenous antibiotic therapy, patients with *A. faecalis* infection will typically experience a positive treatment outcome.

## Supplementary Information

**Additional file 1.**

## Data Availability

The raw data available upon reasonable request from the corresponding authors.

## Abbreviations

AMP: Ampicillin
SAM: Ampicillin-sulbactam
AMX: Amoxicillin
AMC: Amoxycillin-clavulanic acid
CFZ: Cefazolin
CMZ: Cefmetazole
CXM: Cefuroxime
CRO: Ceftriaxone
CTX: Cefotaxime
CTB: Ceftibuten
CAZ: Ceftazidime
FEP: Cefepime
GEN: Gentamicin
AMK: Amikacin
OFX: Ofloxacin
CIP: Ciprofloxacin
MXF: Moxifloxacin
LVX: Levofloxacin
ETP: Ertapenem
IMP: Imipenem
MEM: Meropenem
DOR: Doripenem
TZP: Piperacillin-tazobactam
OXA: Oxacillin
VAN: Vancomycin
TEC: Teicoplanin
CST: Colistin
TGC: Tigecycline
SXT: Trimethoprim-sulfamethoxazole
MDR: Multidrug-resistant
XDR: Extensively drug-resistant
PDR: Pandrug-resistant
ESBL: Extended-spectrum β-lactamase
S: Sensitive
R: Resistant
I: Intermediate
VRE: Vancomycin resistant enterococcus
MSSA: Methicillin sensitive *Staphylococcus aureus*
MRSA: Methicillin resistant *Staphylococcus aureus*
GNB: Gram-negative bacillus
NA: Not accessed
NIL: No data available
